# Pharyngeal Residues Scoring through the Yale Pharyngeal Residue Severity Rating Scale (YPRSRS): Efficacy of Training

**DOI:** 10.1007/s00455-024-10725-y

**Published:** 2024-06-07

**Authors:** Sara Rocca, Luca Negri, Nadia Valenza, Antonio Schindler, Nicole Pizzorni

**Affiliations:** 1 Department of Biomedical and Clinical Sciences, Università degli Studi di Milano, Milan, 20157 Italy; 2 Department of Pathophysiology and Transplantation, Università degli Studi di Milano, Milan, 20122 Italy

**Keywords:** Deglutition disorders, Pharyngeal residues, Fiberoptic endoscopic evaluation of swallowing, Training

## Abstract

The assessment of pharyngeal residues during fiberoptic endoscopic evaluation of swallowing (FEES) is based on visual-perceptual scales that involve clinical subjectivity. Training might be helpful to increase agreement among clinicians. This paper aims to assess the efficacy of training for the assessment of pharyngeal residue in FEES frames and videos through the Yale Pharyngeal Residue Severity Rating Scale (YPRSRS). Twenty-nine clinicians (Phoniatricians, Otorhinolaryngologists, Speech and Language Pathologists) and 47 students in Speech and Language Pathology participated in this study. Fourteen clinicians were randomly allocated to the training group, whilst the remaining 15 served as a control group; all the students participated in the training. Participants scored 30 pairs of videos and frames using the YPRSRS twice, before and after the training for the training groups and at least two weeks apart for the control group. Construct validity, defined as the agreement between each rater and the experts’ scores, and inter-rater reliability were compared among the groups and between the first and the second assessments to verify the efficacy of the training. Construct validity significantly improved at the second assessment in the training group for the pyriform sinuses videos (baseline 0.71 ± 0.04, post-training 0.82 ± 0.05, *p* = .049) and in the students’ group for the valleculae (baseline 0.64 ± 0.02, post-training 0.84 ± 0.02, *p* < .001) and pyriform sinuses videos (baseline 0.55 ± 0.03, post-training 0.77 ± 0.02, *p* < .05). No significant differences were found in the inter-rater reliability in any group. In conclusion, the training seems to improve participants’ agreement with experts in scoring the YPRSRS in FEES videos.

## Introduction

The fiberoptic endoscopic evaluation of swallowing (FEES) is a standard procedure to study swallowing. In 1988, the first FEES report was published by two speech and language pathologists (SLPs) and an Otorhinolaryngologist [1]. Several professionals share the FEES procedure in different countries. In Anglo-American countries, it is predominantly performed by SLPs [2, 3]; in European countries, FEES is mainly performed by Phoniatricians and Otorhinolaryngologists as a medical procedure [3]. Together with the videofluoroscopic study of swallowing (VFSS), FEES is widely considered the gold standard instrumental exam [4] 1. The analyses of FEES recordings allow clinicians to identify signs of impairments in swallow safety (penetration and aspiration) and swallow efficacy (pharyngeal residues) [5].

Various measurement scales are currently available to analyze FEES videos [6]. The assessment of FEES recordings is usually based on visuoperceptual measures; this implies that evaluations of FEES videos are fundamentally subjective. Visuoperceptual scales created for interpreting various signs of dysphagia in FEES are often accompanied by specific training to learn how to use them [7–11]. Neubauer et al. developed the Yale Pharyngeal Severity Rating Scale (YPRSRS) [9], an image-based scale to assess the amount of residue in the valleculae and pyriform sinuses. The severity levels on a 5-point scale are defined “none, trace, mild, moderate, severe” for the valleculae and the pyriform sinuses. An operational description, an anchor image, and a percentage of residue are provided for each point. In a systematic review comparing pharyngeal residue severity rating scales for FEES [12], the YPRSRS was judged to be the most reliable and valid scale. In the original study, 20 raters (Otorhinolaryngology residents, Otorhinolaryngologists, and SLPs) attended a training that included written definitions, visual depictions, explanations, and clarification of doubts [9]. Similarly, a short training on the use of the scale on FEES frames was provided to raters in the validations of the YPRSRS in German [10], Turkish [11], and Italian [13]. The German training [10] lasted 8 min, while the Italian training lasted 4 min [13].

Training in analyzing FEES is a relevant topic, recently reviewed in a scoping sudy [14]. Several post-basic trainings on FEES were designed for exam interpretation and execution by SLPs, Phoniatricians, Otorhinolaryngologists, or other professions [15, 16]. Other training programs targeted different professionals, such as medical student residents [17], neurologists physicians [18], and nurses [15]. The training duration ranged from a minimum of 30 min [18] to over 10 h [19]. A few studies used video-recorded lectures [15] or online lessons [17], and some programs have included self-paced exercises [15, 16, 19], and self-assessments on skills learned [15, 16].

In the validation studies on YPRSRS, the effects of the training was evaluated only on clinicians [9–11, 13], and no comparison was made between ratings of clinical and inexperienced raters such as students. Furthermore, previous papers did not include how to identify and assess pharyngeal residues by using YPRSRS on FEES videos [9–11, 13]. Recently, the YPRSRS has been shown to be reliably used to evaluate pharyngeal residues also in FEES videos, although with average lower reliability coefficients compared to FEES frames [20]. Therefore, training on how to apply the YPRSRS on FEES video is required in order to improve its scoring in these circumstances.

The purpose of the present study was to: (i) develop a training aimed at acquiring skills for the interpretation and evaluation of pharyngeal residue in FEES videos and frames using the YPRSRS scale for medical doctors, SLPs, and students who attend the bachelor’s degree in Speech and Language Pathology; and (ii) verify the training efficacy in improving construct validity and inter-rater reliability. The hypothesis was that training could support participants in developing specific skills in assessing pharyngeal residues in FEES.

## Methods

This project was carried out following the Declaration of Helsinki of the World Medical Association (WHO). Consent of the Ethics Committee of the University of Milan was obtained on 17/11/2020 (number 102/20). Frames and videos used in this work were selected from pre-existing archived material. A randomized controlled trial was conducted among clinicians, whereas a prospective observational pre-post study was performed in students.

### The Yale Pharyngeal Residues Severity Rating Scale

The YPRSRS is an ordinal scale that rates the amount of pharyngeal residues in the valleculae and pyriform sinuses [9]. The definitions of severity are distributed on a 5-point scale (1 = none, 2 = trace, 3 = mild, 4 = moderate, 5 = severe). Each level of the scale corresponds to an operational description, an anchor image, and a percentage of residue. A separate score is provided for the valleculae and the pyriform sinus.

### Selection of the Materials

Seventy pairs of videos and frames with different consistencies were selected from FEES video-recordings collected for previous studies. All FEES examinations were kept anonymous. The FEES were conducted using a XION EF-N flexible fiberscope (XION GmbH, Berlin, Germany) attached to an EndoSTROBE camera (XION GmbH, Berlin, Germany) and recorded as an AVI format. A standard FEES protocol was used including the sequential administration of boluses of thin liquids (5-10-20 ml of blue-dyed water x 3 trials for each volume; International Dysphagia Diet Standardisation Initiative – IDDSI 0; < 50 mPa·s at 50s-1 and 300s-1), pureed food (5-10-20 ml of pudding x 3 trials for each volume; IDDSI 4; 2583.3 ± 10.41 mPa·s at 50s-1 and 697.87 ± 7.84 mPa·s at 300s-1), and regular food (half biscuit x 2 trials; IDDSI 7) [21]. For the present study, only the 5 ml trials were selected for thin liquids and pureed food. For all consistencies, a frame was selected after swallowing the last bolus. As a first step, the 70 pairs of videos and frames were independently assessed by two experienced raters (> 10 years of experience in FEES analysis), a Phoniatrician, and a SLP. Only the pairs of videos and frames that were assigned the same YPRSRS score were selected. Thirty pairs of videos and frames (15 for valleculae, 15 for pyriform sinuses) were selected for for validity and reliability analysis, and an additional 6 pairs (3 for valleculae, 3 for pyriform sinuses) were chosen for the training by consensus. All YPRSRS scores and consistencies of the recorded swallows were included in the analysis.

### Raters

A sample size of 29 clinicians was determined based on the previous studies on the YPRSRS. Inclusion criteria were professional activity as Phoniatricians, Otorhinolaryngologists, SLPs, or resident Otorhinolaryngologists with a minimum clinical experience of 1 year in the dysphagia. In addition, a convenience sample of students who attended the 2nd year of the bachelor’s degree in Speech and Language Pathology was recruited. To participate in the study, all students had to have already attended the classes on dysphagia assessment and treatment.

### Training

Clinicians were randomly allocated to the training or the control group (1:1) based on a random number sequence. Randomization was stratified for the profession (medical doctor vs. SLP) and years of experience (< 5 years vs. 5 ≥ years), according to a previous study [10]. All students received the training.

The training design was based on the characteristics of the trainings for the interpretation of FEES previously described in the literature [15–19, 9, 10]. It consisted of 3 steps, for a total of 160 min. As a first step, the participants viewed a pre-recorded 75-minute video-audio lesson composed of theoretical modules describing dysphagia signs observed during the FEES procedure, pathophysiological mechanisms and consequences of pharyngeal residues, an introduction to the YPRSRS, the clinical application of the scale, and case studies. After the online lesson, each participant independently practiced assigning a YPRSRS score to 6 pairs of frames and videos of FEES (3 for valleculae, 3 for pyriform sinuses), not included in the pre-post training assessment. The results of individual practice and any questions or uncertainties were discussed among participants during a one-hour synchronous meeting moderated by an expert tutor. Debriefing meetings consisted of small groups (4–5 participants) were formed to encourage communication and discussion.

The whole training was delivered using Microsoft Teams (Microsoft Corporation, Redmond, WA).

### Data Collection

Both the training and the control group scored the 30 pairs of videos and frames using the YPRSRS twice: (i) before and after for the training for the training group and the students, at least two weeks between assessments; (ii) at least two weeks between assessments for the control group. The FEES material was submitted for evaluation by the participants through the Google form platform; the order of the videos and frames was randomized for both the first and second assessments. The material was sent together with the scale and the anchor images. All data were treated in a pseudo-anonymized form; each participant was assigned an alphanumeric code. At the end of both assessments, participants completed a self-evaluation questionnaire created ad hoc to investigate participants’ perceived self-efficacy in interpreting FEES with the YPRSRS scale. The self-evaluation questionnaire consisted of 9 items, 2 of which were about anatomical identification of valleculae and pyriform sinuses and 7 items about scoring, based on a 5-point scale (1 = never, 2 = rarely, 3 = sometimes, 4 = often, 5 = always).

### Statistical Analysis

For the analysis, the IBM SPSS v26.0® software for Windows (SPSS Inc, Chicago, Illinois) and the R software v.4.2.0 [22] were used. The clinicians’ and the students’ ratings were analyzed separately.

The baseline characteristics of clinicians were analyzed to make a comparison between the control and training. The Kolmogorov-Smirnov test was used to assess the normality of continuous variables and, as none of them were normally distributed, the Mann-Whitney U test was performed to compare group distributions; frequencies were compared through the chi-square test.

Construct validity and inter-rater reliability were used as a measure for the efficacy of the training. Conversely, intra-rater reliability was not considered a suitable outcome measure to assess the efficacy of the training due to the manipulation introduced by the training itself. Construct validity was defined as the agreement between each rater and the expert score employed as the “gold standard” [9, 10]. Inter-rater reliability was defined as the degree of agreement among raters scoring the same object on the same assessment [23].

Construct validity was calculated through weighted Cohen’s Kappa (quadratic weighting), separately for each rater. The distribution of raters Cohen’s Kappa was compared between the first and the second assessment using the paired t-test. Concerning control, training, and student groups, Cohen’s Kappa distribution was compared separately for the first, and the second assessment, using the one-way analysis of variance (ANOVA) with Tukey HSD adjustment to correct the significance level for post hoc pairwise comparisons.

As for inter-rater reliability, the level of agreement among each group of raters was calculated with Fleiss Kappa (quadratic weighting) both for the first and the second assessment; for each group, indices were subsequently compared using paired sample t-tests based on the linearization method for correlated agreement coefficients [24]. ANOVA with Tukey’s HSD method was also employed to check for differences in Fleiss Kappa values among the three groups separately for the first, and the second assessment.

The benchmark of Landis and Koch was used to evaluate the levels of agreement for the Kappa [25]: 0.00-0.20 slight agreement, 0.21–0.40 fair agreement, 0.41–0.60 moderate agreement, 0.61–0.80 substantial agreement, 0.81-1.00 almost perfect agreement. For the Fleiss Kappa, the following benchmark was adopted: < 0.40 poor, 0.40–0.75 intermediate to good, > 0.75 excellent [26].

To compare the results of the self-assessment questionnaire between the first and the second evaluation within each group, the non-parametric Wilcoxon signed rank test was used. Comparisons among groups for the first and second assessments were performed using Kruskal-Wallis test; pairwise comparisons were adjusted using Bonferroni correction.

## Results

### Raters’ Characteristics

Twenty-nine clinicians were initially recruited for the study. However, 4 clinicians, 2 from the training group and 2 from the control group, dropped out before completing the second assessment. Thus, data from 25 raters were ultimately analyzed. Table 1 shows descriptive statistics on clinicians’ characteristics, together with a comparison between training and control groups. No significant differences were observed between the two groups, except for the higher number of FEES the raters have participated in/performed among the control group (*p* = .02).

**Table 1:** Characteristics of clinicians at baseline: age, sex profession, years of experience, participation in FEES, and execution of FEES are reported

		Clinicians (Nr = 25)	Training (Nr = 12)	Control (Nr = 13)	Groups comparison *p*-value
Age: mean age ± DS		30.12 ± 5.63	27.83 ± 2.98	32.23 ± 6.73	.168^a^
Sex: female n (%)		20 (80.00)	11 (91.67)	9 (69.23)	.820^b^
Profession Speech therapists: n (%)		18 (72.00)	8 (66.67)	10 (76.92)	
Medical doctors: n (%)		7 (28.00)	4 (33.33)	3 (23.08)	.570^b^
Years of experience: <5 year of experience n (%)		15 (60.00)	8 (66.67)	7 (53.85)	
Years of experience: ≥5 year of experience n (%)		10 (40.00)	6 (50.00)	6 (46.15)	.510^b^
N FEES^1^	> 100	9 (36.00)	1 (8.33)	8 (61.54)	
	50–100	10 (40.00)	7 (58.33)	3 (23.08)	
	10–50	6 (24.00)	4 (33.33)	2 (15.38)	.020^b^
Participate regularly at FEES examinations^2^: n (%)		15 (60.00)	9 (75.00)	6 (46.15)	.140^b^
Perform FEES regularly^3^: n (%)		5 (20.00)	3 (25.00)	2(15.38)	.830^b^
Post basic training^4^: n (%)		11 (44.00)	4 (33.33)	7 (53.85)	.300^b^

*NoteNr* = Number of raters; ^1^How many FEES the rater has participated in/performed; ^2^To be present in the room when FEES are being performed; ^3^Only for Medical Doctors: the execution of the FEES through the passage of the fiberscope; ^4^e.g., Postgraduate Diploma, Master program, Ph.D. Groups’ comparisons were performed with the Mann Whitney U Test(a) or the chi-square test (b) according to the variable type

In additions to clinicians, 47 students (22.36 ± 3.54 years, 100% female) also participated in the present study as raters. Overall, 59.57% of the students had never observed a FEES, while 63.83% had performed a university internship with patients with dysphagia.

### Training Results

#### Results

##### Effect of the Training on Construct Validity

Results of the comparison of construct validity values between the first and the second assessment within each group are reported in Table 2. Concerning FEES frames, an almost perfect agreement in the control, training, and students’ groups was observed; no statistically significant differences were found between the first and second evaluations for any of the three groups. As for FEES videos, construct validity values significantly improved between baseline (substantial agreement) and post-training (almost perfect agreement) in the training group for the pyriform sinus videos. In students, the construct validity associated significantly improved in vallecule and pyriform sinus videos: agreement improved from substantial to almost perfect in valleculae videos, and from moderate to almost perfect in the pyriform sinus videos (Table 2).

**Table 2:** Construct validity: comparison between baseline and second assessment in the Training, Control, and Student groups for Valleculae frames and videos, Pyriform sinus frames and videos

	Training (Nr = 12)				Control (Nr = 13)				Students (Nr = 47)			
	1st assessment Cohen’s kappa ± se	2nd assessment Cohen’s kappa ± se	t-test		1st assessment Cohen’s kappa ± se	2nd assessment Cohen’s kappa ± se	t-test		1st assessment Cohen’s kappa ± se t_(df=13)_	2nd assessment Cohen’s kappa ± se	t-test	
			t_(df=11)_	***p***			t_(df=12)_	***p***			t_(df=46)_	***p***
Valleculae frames	0.86 ± 0.03	0.87 ± 0.03	-1,01	0.337	0.92 ± 0.01	0.93 ± 0.01	-0,93	0.369	0.85 ± 0.01	0.87 ± 0.01	-1.58	0.120
Valleculae videos	0.71 ± 0.06	0.83 ± 0.03	-1,78	0.102	0.86 ± 0.04	0.86 ± 0.04	-0,159	0.876	0.64 ± 0.02	0.84 ± 0.02	-7.70	**< 0.001**
Pyriform sinus frames	0.84 ± 0.03	0.79 ± 0.05	1,39	0.096	0.89 ± 0.01	0.84 ± 0.04	1.77	0.102	0.85 ± 0.01	0.84 ± 0.01	0.93	0.357
Pyriform sinus videos	0.71 ± 0.04	0.82 ± 0.05	-2,21	**0.049**	0.92 ± 0.01	0.93 ± 0.01	-0,93	0.369	0.55 ± 0.03	0.77 ± 0.02	-8.36	**< 0.001**

*NoteNr* = Numbers of raters, Significant p-values are reported in bold

Based on a rating of 30 frames and videos

Pairwise comparisons of construct validity values among groups are reported in Table 3. At the baseline, the ANOVA post-hoc analysis showed that students exhibited significantly lower values of construct validity compared to the control group for the valleculae (both frames and video) and compared to both groups of clinicians for the pyriform sinus videos (Table 3). At the second assessment, students had lower values of construct validity compared to the control group only for the assessments of the valleculae frames (Table 4). No significant differences in construct validity scores were found between the training and control groups.

**Table 3:** Construct validity: Pairwise comparisons among Training, Control, and Students groups for Valleculae frames and videos, Pyriform sinus frames, and videos: ANOVA results

	1st assessment				2nd assessment			
	F (2,69) *p*-value	Training VS Control Δm; ± se Sig.	Training VS Students Δm; ± se Sig.	Students VS Control Δm; ± se Sig.	F (2,69) *p*-value	Training VS Control Δm; ± se Sig.	Training VS Students Δm; ± se Sig.	Students VS Control Δm; ± se Sig.
Valleculae frames	3.94 *p* = .240	-0.06 ± 0.03 0.097	0.002 ± 0.02 0.997	**-0.06 ± 0.02** **0.020**	3.17 *p* = .048	-06 ± 0.03 0.166	0.003 ± 0.03 0.989	**-0.06 ± 0.03** **0.040**
Valleculae videos	8.97 *p* < .001	-0.14; ±0.06 0.077	-0.07; ±0.05 0.375	**-0.22; ±0.05** **< 0.001**	0.22 *p* = .810	-	-	-
Pyriform sinuses frames	0.97 *p* = .380	-	-	-	0.79 *p* = .460	-	-	-
Pyriform sinus videos	9.88 *p* < .001	-0.07 ± 0.07 0.599	**0.15 ± 0.06** **0.0240**	**-0.23 ± 0.06** **< 0.001**	0.633 *p* = .530	-	-	-

*Note* Tukey HSD adjusted comparisons, Significant p-values are reported in bold

**Table 4:** Inter-rater reliability: comparison between baseline and second assessment in the Training, Control, and Student groups for Valleculae frames and videos, Pyriform sinus frames and videos

	Control (Nr = 13)				Training (Nr = 12)				Students (Nr = 47)			
	1st assessment Fleiss K ± se	2nd assessment Fleiss K ± se	t-test		Pre training Fleiss K ± se	Post training Fleiss K ± se	t-test		Pre training Fleiss K ± se	Post training Fleiss K ± se	t-test	
			t_(df=14)_	***p***			t_(df=14)_	***p***			t_(df=14)_	***p***
Valleculae frames	0.89 ± 0.04	0.92 ± 0.03	1.43	0.173	0.80 ± 0.05	0.80 ± 0.05	-0.11	0.915	0.81 ± 0.06	0.83 ± 0.05	0.97	0.345
Valleculae videos	0.78 ± 0.06	0.80 ± 0.10	0.30	0.767	0.63 ± 0.11	0.76 ± 0.06	1.06	0.307	0.67 ± 0.08	0.78 ± 0.05	1.93	0.073
Pyriform sinus frames	0.85 ± 0.05	0.75 ± 0.08	-1.50	0.155	0.77 ± 0.07	0.70 ± 0.08	-1.39	0.184	0.81 ± 0.05	0.81 ± 0.05	0.55	0.593
Pyriform sinus videos	0.72 ± 0.08	0.67 ± 0.9	-1.06	0.308	0.59 ± 0.11	0.71 ± 0.07	1.62	0.126	0.54 ± 0.09	0.68 ± 0.08	1.81	0.091

*Note Nr* = Numbers of raters; Significant p-values are reported in bold; Based on a rating of 30 frames and videos

##### Effect of the Training on Inter-Rater Reliability

Table 4 shows inter-rater reliability results. No significant differences were observed between the first and the second assessments. Moreover, no significant differences in the inter-rater reliability values were found among groups at any time point (Table 5).

**Table 5:** Inter-rater reliability: comparison among Training, Control, and Student groups for Valleculae frames and videos, Pyriform sinus frames and videos: ANOVA results

	1st assessment	2nd assessment
	F (2,69) *p*-value	F (2,69) *p*-value
Valleculae frames	0.232 *p* = .793	0.552 *p* = .578
Valleculae videos	0.350 *p* = .706	0.05 *p* = .950
Pyriform sinuses frames	0.186 *p* = .830	0.615 *p* = .540
Pyriform sinus videos	0.487 *p* = .617	0.024 *p* = .976

### Self-Assessment

Self-assessment results on the perceived self-efficacy in interpreting FEES with the YPRSRS scale are reported in Tables 6 and 7. The Wilcoxon signed-rank test found no significant difference between the first and the second assessments among clinicians, regardless of their training status. Students’ group values significantly improved at the second assessment (Table 6), although the perceived self-efficacy remained lower than clinicians’ (Table 7).

**Table 6:** Self-assessment: comparisons between baseline and second assessment in the Training, Control, and Student groups for the 9 items of the self-assessment questionnaire

	Control (Nr = 13)				Training (Nr = 12)				Students (Nr = 46)			
	1st assessment median (IQR)	2nd assessment median (IQR)	Wilcoxon signed rank test		Pre training median (IQR)	Post training median (IQR)	Wilcoxon signed rank test		Pre training median (IQR)	Post training median (IQR)	Wilcoxon signed rank test	
			Z value	*p*			Z value	*p*			Z value	*p*
1. I felt confident in identifying valleculae in FEES frames and videos	5 (4–5)	5 (4–5)	0.000	1.000	5 (5–5)	5 (4–5)	-0.447	0.655	4 (4–5)	5 (4–5)	-2.874	**0.004**
2. I felt confident in identifying pyriform sinuses in FEES frames and videos.	5 (4–5)	5 (4–5)	-0.577	0.564	5 (4–5)	5 (4.25-5)	-1.342	0.180	4 (4–5)	5 (4–5)	-3.152	**0.002**
3. I felt confident in scoring YPRSRS for pharyngeal residues in valleculae in FEES frames and videos.	4 (3.5-4)	4 (4–4)	-1.732	0.083	4 (3,25 − 4)	4 (4–4)	-1.732	0.083	3 3–3)	4 (3–4)	-4.564	**< 0.001**
4. I felt confident in scoring YPRSRS for pharyngeal residues in pyriform sinuses in FEES frames and videos.	4 (3–4)	4 (3.5-4)	-1.732	0.083	3.50 (3–4)	4 (3.25-4)	-0.816	0.414	3 (3–3)	3 (3–4)	-3.788	**< 0.001**
5. I felt confident in scoring YPRSRS for pharyngeal residues with liquids	3 (3–4)	3 (3–4)	-0.447	0.655	3 (2.25-4)	3 (3–4)	-0.378	0.705	2.5 (2–3)	3 (2–3)	-2.128	**0.033**
6. I felt confident in scoring YPRSRS for pharyngeal remnants with pureed foods	4 (4-4.5)	4 (4-4.5)	-0.816	0.414	4 (4–4)	4 (4–4)	-1.414	0.157	3 (3–4)	4 (4–4)	-4.372	**< 0.001**
7. I felt confident in scoring YPRSRS for pharyngeal remnants with solid foods	4 (3–4)	4 (4–4)	-1.342	0.180	4 (4–4)	4 (4–4)	-0.447	0.655	3 (3–4)	3.5 (3–4)	-2.682	**0.007**
8. In the videos, I felt confident in selecting the timing of evaluating pharyngeal residues.	4 (3.5–4.5)	4 (3.5-5)	-1.000	0.317	3.50 (3–5)	4 (3.25-5)	-1.190	0.234	2 (2–3)	4 (4–5)	-5-506	**< 0.001**
9. I needed to watch each FEES video several times before assigning a YPRSRS score.	3 (3-3.5)	3 (2.5-3)	-1.134	0.257	3 (2–4)	4 (3-4.75)	-1.897	0.058	4 (3–4)	3 (3–3)	-2.966	**0.003**

*NoteNr* = Numbers of raters, significant p-values are reported in bold

**Table 7:** Self-assessment: pairwise comparisons among No training, Training, and Students groups for the 9 items of the self-assessment questionnaire

	Baseline				2nd assessment			
	H(2) *p*-value	Training VS Control Δm; ± se Sig.	Training VS Students Δm; ± se Sig.	Students VS Control Δm; ± se Sig.	H(2) *p*-value	Training VS Control Δm; ± se Sig.	Training VS Students Δm; ± se Sig.	Students VS Control Δm; ± se Sig.
1. I felt confident in identifying valleculae in FEES frames and videos	11.848 *p* = .003	0.702	**0.004**	**0.013**	0.498 *p* = .780	0.903	0.649	0.532
2. I felt confident in identifying pyriform sinuses in FEES frames and videos.	8.147 *p* = .017	0.717	**0.053**	**0.014**	1.621 *p* = .445	0.445	0.204	0.735
3. I felt confident in scoring YPRSRS for pharyngeal residues in valleculae in FEES frames and videos.	21.659 *p* < .001	0.937	**< 0.001**	**< 0.001**	11.977 *p* = .003	1.000	**0.008**	**0.006**
4. I felt confident in scoring YPRSRS for pharyngeal residues in pyriform sinuses in FEES frames and videos.	15.424 *p* < .001	0.960	**0.003**	**0.002**	9.138 *p* = .010	0.928	**0.024**	**0.014**
5. I felt confident in scoring YPRSRS for pharyngeal residues with liquids	12.132 *p* = .002	0.535	**0.026**	**0.002**	7.034 *p* = .030	0.945	**0.038**	**0.040**
6. I felt confident in scoring YPRSRS for pharyngeal remnants with pureed foods	17.903 *p* < .001	0.932	**0.001**	**< 0.001**	7.960 *p* = .019	0.756	0.051	**0.016**
7. I felt confident in scoring YPRSRS for pharyngeal remnants with solid foods	16.868 *p* < .001	0.404	**< 0.001**	**0.008**	10.650 *p* = .005	0.867	**0.007**	**0.017**
8. In the videos, I felt confident in selecting the timing of evaluating pharyngeal residues.	24.996 *p* < .001	0.768	**< 0.001**	**< 0.001**	0.008 *p* = .996	0.998	0.931	0.947
9. I needed to watch each FEES video several times before assigning a YPRSRS score.	3.209 *p* = .201	0.223	0.822	0.123	5.229 *P* = .073	**0.039**	**0.038**	0.602

*Note* significant p-values are reported in bold

## Discussion

This study examined the training efficacy in improving the raters’ performance by assessing pharyngeal residue in FEES videos and frames using the YPRSRS and self-assessment changes from baseline and second assessment. The results obtained showed an improvement in agreement between participants, particularly students, and the experts in interpreting FEES videos. This improvement cannot solely be attributed to task repetition.

Our training spanned approximately 160 min, encompassing video lessons, independent practice, and a debriefing meeting. Unlike previous trainings for the use of the YPRSRS [9–11, 13], our current study introduced both practical exercises and a debriefing meeting to complement the theoretical lessons. Notably, the presented training lasted nearly 3 h, a substantial increase compared to the previous studies (8 min in the German study [10] and 4 min in the Italian study [13]). Other training programs on visuoperceptual scales typically have a similar or longer duration compared to the current one. In Kaneoka et al.‘s study [8], which aimed to demonstrate the reliability and validity of The Boston Residue and Clearance Scale (BRACS), four speech-language pathologists (SLPs) participated in a 3-hour session led by an expert clinician and co-creator of BRACS. The Visual Analysis of Swallowing Efficiency and Safety (VASES) training [7] comprised five parts covering VASES rules, practice with five FEES videos, a pre-recorded 60-minute session, additional practice with another five FEES videos, and a live 60-minute session. The median completion time for this training was 6 h.

Furthermore, previous studies that validated the YPRSRS in different languages [9–11, 13] analyzed the effectiveness of the training in frames but not in videos. Videos were included in this study because they better reflect the dysphagia assessment in the actual clinical practice. In addition, the video application is preferred in the instrumental evaluation of swallowing to ensure the quality of diagnosis and management of dysphagia [27]. The training presented in this study has advantages and disadvantages. The online mode allowed participants to take classes from home at the times most convenient for them. Online and face-to-face training are comparable in terms of effectiveness [17]. However, the asynchronous mode did not allow direct and timely exchange between instructors and participants. This disadvantage was partly offset by the debriefing.

Concerning clinicians, construct validity for pyriform sinus videos significantly improved after training, while no improvement emerged from the construct validity analysis of the control group in the second assessment. No group showed significant differences in inter-rater reliability values between the baseline and second assessments. Despite this, in the “training” group, it is possible to consider a trend of improvement in the valleculae videos (kappa values ranged from an intermediate to good agreement to an excellent agreement). The limited sample size, based on previously published studies [10], could have led to a lack of statistical power to detect significant differences. The training seems to have improved the rating precision of clinicians in assessing videos; however, similarly to previous studies [9–11, 13], such a result was not observed in raters’ performance during frames baseline evaluation.

Differently from previous studies, no difference was found between training and control groups for inter-rater reliability scores associated with frames assessment: Neubauer et al. [9] reported a significantly higher value for inter-rater reliability of trained raters in frames for both locations and Gerschke et al. [10] found significant differences between trained and untrained raters only for valleculae frames ratings. To date, the YPRSRS is widely employed, although it was newly devoloped or recently validated when the cited studies were conducted [9, 10]. Thus, it is possible to assume that participants were still not familiar with the scale in previous studies [10, 11, 13], while the higher level of clinical experience with the scale from participants in the present study could have influenced the efficacy of the training for frames.

In the students’ group, values for construct validity in FEES videos were significantly higher after training; the training reduced the gap between students’ and clinicians’ accuracy at the baseline as students achieved values of construct validity similar to clinicians. For the first time, students were selected to participate in training on the YPRSRS. The present results seem to confirm that the training, especially on less experienced raters, improves the accuracy of pharyngeal residues assessment in videos.

Self-questionnaire analyses of efficacy showed that students felt more confident using the scale after the training. This improvement in self-efficacy aligns with the progress noted in the second-assessment analysis, particularly in the pairwise comparisons with clinicians. No significant differences were observed between the baseline and second assessments in the clinicians’ groups. Notably, students, in general, exhibited lower confidence in applying the scale compared to clinicians in pairwise comparisons. An interesting finding emerges when comparing the training group to both the control and student groups during the second assessment regarding item 9. Item 9 involves the necessity of reviewing the video multiple times before assigning a score. The training likely enhanced clinicians’ thoroughness; consequently, they may have found it beneficial to watch the videos several times before finalizing their scores. Self-assessment questionnaires were not present in previous studies [9–11]. Moreover, none of the studies mentioned in a recent review on training to analyze functional parameters with FEES [14] included a measure specifically addressing self-confidence [18, 7–10, 28]. Investigating this aspect assumes relevance because, especially in inexperienced trainees such as students, good self-assessment skills are considered a key component in developing clinical skills [29].

There are some limitations in this study. All the students participated in the training. Thus, having a control group composed of untrained students was impossible. Future studies should also include a control group of students. Because of drop-outs, the number of raters who completed the second assessment was less than the expected sample size. Moreover, this study differs from previous studies on the YPRSRS as the “best-of-the-best” criterion was not used in choosing frames and videos. Although it may better reflect what clinicians experience in clinical practice, having included videos gathered from everyday clinical practice in a University Hospital may have made the evaluation more complex.

## Conclusion

The results showed that the training can improve clinicians’ and students’ agreement with experts in assessing pharyngeal residues with the YPRSRS in the FEES videos. Promoting evidence-based training courses would allow students and clinicians with different backgrounds to have a standard method of interpreting FEES and sharing information in clinical practice and research.

## Data Availability

The data that support the findings of this study are available from the corresponding author upon reasonable request.
